# Single Cell FRET Analysis for the Identification of Optimal FRET-Pairs in *Bacillus subtilis* Using a Prototype MEM-FLIM System

**DOI:** 10.1371/journal.pone.0123239

**Published:** 2015-04-17

**Authors:** Ruud G. J. Detert Oude Weme, Ákos T. Kovács, Sander J. G. de Jong, Jan-Willem Veening, Jeroen Siebring, Oscar P. Kuipers

**Affiliations:** 1 Molecular Genetics Group, Groningen Biomolecular Sciences and Biotechnology Institute, Centre for Synthetic Biology, University of Groningen, 9747 AG Groningen, The Netherlands; 2 Lambert Instruments B.V., Oosteinde 16, 9301 ZP Roden, The Netherlands; CNR, ITALY

## Abstract

Protein-protein interactions can be studied *in vitro*, e.g. with bacterial or yeast two-hybrid systems or surface plasmon resonance. In contrast to *in vitro* techniques, *in vivo* studies of protein-protein interactions allow examination of spatial and temporal behavior of such interactions in their native environment. One approach to study protein-protein interactions *in vivo* is via Förster Resonance Energy Transfer (FRET). Here, FRET efficiency of selected FRET-pairs was studied at the single cell level using sensitized emission and Frequency Domain-Fluorescence Lifetime Imaging Microscopy (FD-FLIM). For FRET-FLIM, a prototype Modulated Electron-Multiplied FLIM system was used, which is, to the best of our knowledge, the first account of Frequency Domain FLIM to analyze FRET in single bacterial cells. To perform FRET-FLIM, we first determined and benchmarked the best fluorescent protein-pair for FRET in *Bacillus subtilis* using a novel BglBrick-compatible integration vector. We show that GFP-tagRFP is an excellent donor-acceptor pair for *B*. *subtilis in vivo* FRET studies. As a proof of concept, selected donor and acceptor fluorescent proteins were fused using a linker that contained a tobacco etch virus (TEV)-protease recognition sequence. Induction of TEV-protease results in loss of FRET efficiency and increase in fluorescence lifetime. The loss of FRET efficiency after TEV induction can be followed in time in single cells via time-lapse microscopy. This work will facilitate future studies of *in vivo* dynamics of protein complexes in single *B*. *subtilis* cells.

## Introduction

Bacteria have long been regarded as vesicles filled with proteins without any internal organization. However, the cytosol of bacterial cells is densely crowded [1], so a high level of organization is expected to ensure proper functioning of cellular processes. Recently, there has been growing interest in elucidating potential spatial organization inside bacterial cells. A large body of work in bacteria has now revealed spatial organization for DNA-protein interactions, protein localization, and protein-protein interactions [2–5]. Protein-protein interactions during cell division, regulatory interactions, and metabolic processes are increasingly studied.

Previously, techniques for studying protein-protein interactions were either indirect (e.g. yeast- or bacterial two hybrid) or *in vitro* methods (e.g. Surface Plasmon Resonance). These techniques are practical for screening potential interaction partners or to study binding affinities. In order to gain insight into the *in vivo* dynamics of these interactions, the *in vivo* method needs to produce time-resolved information. Förster Resonance Energy Transfer (FRET) allows time-resolved inspection of protein-protein interactions. Single cell FRET analysis allows for the investigation of individual differences of protein-protein interactions, rather than studying an average FRET efficiency of a population.

Fluorescence microscopy only allows identification of protein co-localization due to its limited resolution. FRET is the non-radiative energy transfer from an excited donor fluorophore to an acceptor fluorophore that can only occur when donor and acceptor are in very close vicinity of each other (<10 nm). FRET is therefore a useful tool for proving interactions via excitation of the donor fluorophore and measuring emission of the acceptor molecule [6–8].

FRET was first described by Förster [9–11] and found widespread use in molecular biology [12,13] ever since the introduction of various fluorescent proteins (FPs)[14]. FRET has been successfully applied to demonstrate interactions between proteins, e.g. to study the assembly of the divisome in *Escherichia coli* [15], and the composition of the *Bacillus subtilis* competence machinery [16]. For a successful FRET experiment there are three requirements. First, the donor and acceptor fluorophores are within 1–10 nm from each other. Second, the donor emission spectrum overlaps with the acceptor excitation spectrum, and third, the fluorophores have similar orientation of the dipoles [11]. While the spectral overlap is necessary, it is also a disadvantage, because the acceptor can be excited by the light used to excite the donor instead of getting excited by non-radiative energy transfer. Also the donor emission can pass the acceptor emission filter. The potential of donor and acceptor bleedthrough demands corrections via imaging of samples with only a donor or acceptor fluorophore [6]. Another way to overcome the bleedthrough problems caused by spectral overlap is measuring the fluorescence lifetime of the donor fluorophore in presence or absence of an acceptor. A population of excited fluorophores displays a characteristic decay of spontaneous emission.

In this study the FRET efficiency was determined in two ways: the first method is via the detection of sensitized emission [6]: the measurement of the acceptor emission that originates from the resonance energy transfer from the excited donor. The second method is by measuring fluorescence lifetime, which is defined as the time needed for the fluorescence intensity to decrease to 1/e (approx. 37%) of the initial intensity immediately after excitation [17] and is commonly in the nanosecond range. Anything that quenches fluorescence – offers (more) non-radiative decay options, such as FRET does – will decrease the fluorescence lifetime [18].

Frequency Domain Fluorescence Lifetime Imaging Microscopy (FD-FLIM) [11,17–19] allows wide-field fluorescence lifetime determination via phase modulation [17]. For FD-FLIM the excitation light is modulated, consequently resulting in a modulated emission signal. The lifetime of the fluorophore studied causes a delay in the phase of the modulated emission compared to the phase of the modulated excitation. By modulating the sensitivity of the detector at the same frequency as the excitation signal, the phase delay between emission and excitation can be measured and a fluorescence lifetime can be calculated in every pixel of the image. FD-FLIM allows fast wide-field fluorescence lifetime acquisitions and is highly suitable for time-lapse microscopy and thus time resolved FRET analysis. See Zhao *et al*. [20] and the textbook of Lakowicz [17] for a detailed explanation of FD-FLIM.

Here, we investigated which FRET couple is best suited for dynamic protein-protein interaction studies in single cells of the Gram-positive model bacterium *B*. *subtilis* [21]. Several genes coding for fluorescent proteins with potential good FRET properties were cloned pairwise, integrated at the *amyE* locus, expressed, and tested for FRET properties. The FPs were covalently linked with a linker containing a TEV protease recognition sequence. An inducible TEV protease gene was co-cloned with the FRET-pair allowing conditional high/low FRET efficiency situations. FRET efficiency was determined via sensitized emission and via a prototype MEM-FLIM system. The currently available FD-FLIM systems make use of an image intensifier, of which the photocathode is modulated at high frequencies (MHz). The image intensifier is typically the limiting factor for the spatial resolution, and is susceptible to damage by high light intensities. Therefore, FLIM recording is difficult to automate and is vulnerable. The prototype MEM-FLIM system used here modulates the CCD-sensor of the detector directly at the pixel level [20] resulting in a wide-field FLIM system that can easily be integrated in automated microscopy set-ups for time-lapse microscopy to study dynamics of protein-protein interactions. Furthermore, and more importantly, the increased spatial resolution is sufficient for single bacterial cell FRET-FLIM. To our knowledge this is the first study that incorporates single bacterial cell FD-FLIM analysis on a wide-field microscope.

## Materials and Methods

### Bacterial strains, plasmids, oligonucleotides, and growth conditions

The strains, plasmids, and oligonucleotides used in this study are listed in Table 1, Table 2, and Table 3 respectively. *Escherichia coli* MC1061 was used for cloning. All strains were cultivated on LB (Lysogeny Broth) medium at 37°C, supplemented with 100 μg/ml ampicillin or 100 μg/ml spectinomycin when appropriate. For the time-lapse experiment a chemically defined medium was used.

**Table 1:** The strains used in this study.

*Strain*	Genotype	Source or reference
*E*. *coli*		
*MC1061*	F^–^ *araD139* Δ*(ara-leu)7696 galE15 galK16* Δ*(lac)X74 hsdR2* (r_K_^–^m_K_^+^) *mcrA mcrB1 rpsL* (Str^r^)	Laboratory stock
*B*. *subtilis*		
*BSG1004*	*B*. *subtilis* ΔscpA and thrC::TEV-protease	[32]
*168*	*trpC2*	Bacillus Genetic Stock Center
*DOW01*	*168* with *thrC*::*P*_*xyl*_ *-TEV-protease* ery-linc^r^	This study
*DOW03*	*DOW01* with *amyE*::*P*_*hyper-spank*_ *-Cerulean* Spec^r^	This study
*DOW05*	*DOW01* with *amyE*::*P*_*hyper-spank*_ *-GFP* Spec^r^	This study
*DOW09*	*DOW01* with *amyE*::*P*_*hyper-spank*_ *-Venus* Spec^r^	This study
*DOW10*	*DOW01* with *amyE*::*P*_*hyper-spank*_ *-mCherry* Spec^r^	This study
*DOW13*	*DOW01* with *amyE*::*P*_*hyper-spank*_ *-tagRFP* Spec^r^	This study
*DOW14*	*DOW01* with *amyE*::*P*_*hyper-spank*_ *-mKate2* Spec^r^	This study
*DOW16*	*DOW01* with *amyE*::*P*_*hyper-spank*_ *-Cerulean-Venus* Spec^r^	This study
*DOW21*	*DOW01* with *amyE*::*P*_*hyper-spank*_ *-GFP-mCherry* Spec^r^	This study
*DOW23*	*DOW01* with *amyE*::*P*_*hyper-spank*_ *-GFP-tagRFP* Spec^r^	This study
*DOW24*	*DOW01* with *amyE*::*P*_*hyper-spank*_ *-GFP-mKate2* Spec^r^	This study
*DOW26*	*DOW01* with *amyE*::*P*_*hyper-spank*_ *-Venus-mCherry* Spec^r^	This study

**Table 2:** The plasmids used in this study.

Plasmid	Genotype	Source or reference
pUC18	Amp^r^ *lacZ’*	NCBI accession L09136
pDG1664	Amp^r^ *‘thrC Ery* ^*r*^ *-linc* ^*r*^ *thrC’*	[45]
pDR111	Amp^r^ *amyE’ Spec* ^*r*^ *lacI P*_*hyper-spank*_ *amyE*	D. Rudner
pDOW01	*amyE´* Spec^r^ *lacI P*_*hyper-spank*_ *amyE*	This study
pDOW03	*amyE´* Spec^r^ *lacI P*_*hyper-spank*_ *-Cerulean amyE*	This study
pDOW05	*amyE´* Spec^r^ *lacI P*_*hyper-spank*_ *-GFP amyE*	This study
pDOW09	*amyE´* Spec^r^ *lacI P*_*hyper-spank*_ *-Venus amyE*	This study
pDOW10	*amyE´* Spec^r^ *lacI P*_*hyper-spank*_ *-mCherry amyE*	This study
pDOW13	*amyE´* Spec^r^ *lacI P*_*hyper-spank*_ *-tagRFP amyE*	This study
pDOW14	*amyE´* Spec^r^ *lacI P*_*hyper-spank*_ *-mKate2 amyE*	This study
pDOW16	*amyE´* Spec^r^ *lacI P*_*hyper-spank*_ *-Cerulean-Venus amyE*	This study
pDOW21	*amyE´* Spec^r^ *lacI P*_*hyper-spank*_ *-GFP-mCherry amyE*	This study
pDOW23	*amyE´* Spec^r^ *lacI P*_*hyper-spank*_ *-GFP-tagRFP amyE*	This study
pDOW24	*amyE´* Spec^r^ *lacI P*_*hyper-spank*_ *-GFP-mKate2 amyE*	This study
pDOW26	*amyE´* Spec^r^ *lacI P*_*hyper-spank*_ *-Venus-mCherry amyE*	This study

**Table 3:** The oligonucleotides used in this study.

Oligonucleotide	Sequence (5’ -> 3’)
pDR111_*amyE*_FW *Nco*I	TGATG*CCATGG*AATCAAATAAGGAGTGTCAAGAATG
pDR111_*amyE*_REV *Spe*I	TTGCT*ACTAGT*CGTCTAGCCTTGCCCTCAATG
pUC18_ORI_FW *Spe*I	TGAGG*ACTAGT*GTACCACCCCGTAGAAAAGATCAAAG
pUC18_ORI_REV *Nco*I	GGATA*CCATGG*CTCGGGATAACGCAGGAAAGAAC
pDR111_quikchange_FW	GACCGGCGCTCAGAATCCTAACTCAC
pDR111_quikchange_REV	GTGAGTTAGGATTCTGAGCGCCGGTC
pDOW_seq1	CATGGCTCGGGATAACGCAGGAAAG
pDOW_seq2	CTGATTCTGACCGGGCACTTGGG
pDOW_seq3	CAAATAAAGCACTCCCGCGATC
pDOW_seq4	GATCTGTCAATGGTTCAGATAC
pDOW_seq5	TCTAGAGCTGCCTGCCGCGTTTCG
pDOW_seq6	ACGCGCCGTCGCAAATTGTC
pDOW_seq7	AATCAGCTGTTGCCCGTCTCAC
pDOW_seqINSERT_FW	CTACAAGGTGTGGCATAATGTG
pDOW_seqINSERT_REV	AGCTTGCATGCGGCTAGCTG
Cerulean/Venus_FW_BglBrick	GAGCTCGAATTCATGAGATCTATGTCAAAAGGAGAAGAACTTTTTAC
Cerulean/Venus_REV_BglBrick	GTCGAGCTCGAGTAAGGATCCTTTATAAAGTTCGTCCATACC
sfGFP_FW BglBrick	GAGCTCGAATTCATGAGATCTATGTCAAAGGGAGAAGAATTG
sfGFP_REV BglBrick	GTCGAGCTCGAGTAAGGATCCCTTATAAAGTTCATCCATTCCGTGTGTGATTC
mCherryDSM_FW BglBrick	GAGCTCGAATTCATGAGATCTATGAGCAAAGGAGAAGAAG
mCherryDSM_REV BglBrick	GTCGAGCTCGAGTAAGGATCCTTTGTAAAGCTCATCCATTC
tagRFP_FW BglBrick	GAGCTCGAATTCATGAGATCTATGTCAGAACTTATCAAGGAAAATATG
tagRFP_REV BglBrick	GTCGAGCTCGAGTAAGGATCCTTTATGTCCCAATTTACTAGG
mKate2_FW BglBrick	GAGCTCGAATTCATGAGATCTATGTCAGAACTTATCAAGGAAAATATG
mKate2_REV BglBrick	GTCGAGCTCGAGTAAGGATCCACGGTGTCCCAATTTAC
constructTEV-FW+*EcoR*I	GCTGTCAAACATGAGAATTC
constructTEV-REV+*Bgl*II	CGCGAGATCTGGATTTCCTTACGCGAAATACG

Restriction sites are shown in italics. BglBrick prefix in forward (FW) primer and suffix in reverse (REV) primer are underlined.

### Recombinant DNA techniques

DNA purification, restriction and ligation were done as described before [22]. Fast Digest Restriction enzymes, Phusion DNA polymerase and T4 DNA ligase were obtained from Fermentas (St. Leon-Rot, Germany). Synthetic DNA was ordered from Mr. Gene (Regensburg, Germany).

### Plasmid construction

In this study, a new *B*. *subtilis* integration vector, pDOW01, was constructed by modifying pDR111 (kind gift of David Rudner). Both vectors integrate chromosomally in the *amyE* locus. The BglBrick assembly standard [23] was introduced into pDR111 resulting in pDOW01 to facilitate easy cloning of biological parts.

Further, a TEV protease recognition site (ENLYFQG) coding sequence was inserted as a linker in the middle of the BglBrick cloning site. The BglBrick cloning site, with *EcoR*I, *Bgl*II, *BamH*I and *Xho*I restriction sites, always remains present during cloning to realize up- or downstream insertion of a new part into an existing construct. The BglBrick cloning strategy is adapted from the BioBrick cloning method [24].

To create pDOW01 from pDR111 the following changes were made: the ORI for *E*. *coli* was replaced with the ORI for *E*. *coli* from pUC18 to remove the *Bgl*II and *Xho*I restriction sites, the Amp^R^ was removed, the BglBrick [23] restriction sites are introduced for cloning, and a TEV-protease recognition site was introduced in the middle of the BglBrick cloning site.

**Fig 1:**
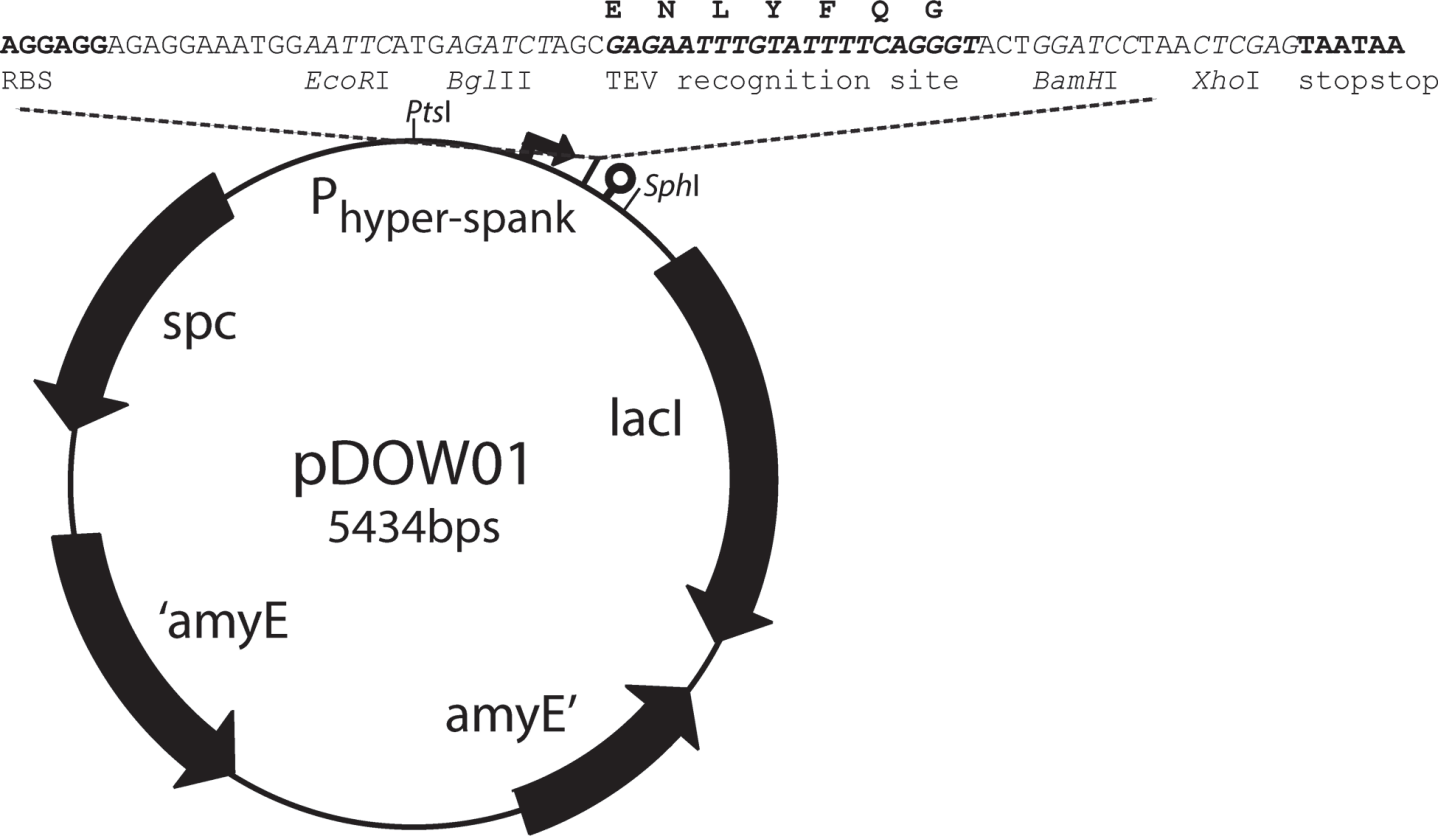
Map of the *amyE* integration vector pDOW01 with BglBrick cloning site, *EcoR*I, *Bgl*II, *BamH*I and *Xho*I indicated in italics. Indicated in bold are the RBS (AGGAGG), the TEV-protease recognition site (GAGAATTTGTATTTTCAGGGT; amino acid sequence ENLYFQG) and the two stopcodons (TAATAA).

The homologous regions of the *amyE* gene and the spectinomycin marker from pDR111 (from 115–4707) and the *E*. *coli* origin of replication (1888–2583) from the pUC18 vector were PCR amplified including the *Nco*I and *Spe*I restriction sites in the primer sequences to combine the fragments into a pDR-pUC-hybrid (see Table 3 for the primers). Subsequently, Quikchange PCR (Agilent) was used to remove the *BamH*I site at position 745, with primers pDR111_quikchange_FW and pDR111_quikchange_REV. The part between 2100–2528 bp in the original pDR111 vector was redesigned *in silico* and ordered from Mr. Gene (Regensburg, Germany), with the following changes. The *Xho*I, *Bgl*II and *EcoR*I sites were removed by point mutation. The *Sal*I site was removed and in the upstream direction a RBS with a seven bp spacer to the start codon was inserted. The start codon was followed by the BglBrick prefix, the TEV-protease recognition site, the BglBrick suffix, two stop codons, and a strong terminator sequence. The IPTG-inducible P_hyper-spank_ promoter from pDR111 remained unchanged. The synthetic DNA was inserted into the pDR-pUC-hybrid by replacing the original 422 bp between the *Sph*I- and *Pst*I-sites with the 529 bp synthetic DNA via restriction and ligation. This final cloning step resulted in our basic cloning vector, pDOW01 (Fig 1). The newly constructed plasmid was fully re-sequenced and the plasmid sequence has been submitted to the Genbank database under accession number KM009065.

### Plasmid construction – fluorophore insertion

The fluorescent proteins used in this study are: Cerulean (a cyan FP [25]), Venus (a yellow FP [26]), sfGFP(Sp) [27], mCherry (a red FP [28], which is codon optimized by DSM as described before [27]), mKate2 (a far-red FP, Evrogen) and tagRFP (a red-orange FP, Evrogen). Codon optimization was done for sfGFP, mCherry, mKate2 and tagRFP as described before [27].

pDOW01 was used as basic vector to construct the following FRET pairs and their donor and acceptor only counterparts: Cerulean-Venus, GFP-mCherry, GFP-mKate2, GFP-tagRFP, and Venus-mCherry. Cerulean and Venus are improved versions of CFP and YFP [25,26]. The pairs were selected for use as a FRET pair based on the spectral properties [29] (see also: http://www.microscopyu.com/).

All genes encoding for fluorescent proteins were amplified by PCR with the BglBrick prefix (*EcoR*I, *Bgl*II) in the forward primer and the BglBrick suffix (*BamH*I, *Xho*I) in the reverse primer for insertion into pDOW01 (see Table 3). Sequencing was used to verify the construct sequences.

Two fluorophores were linked to each other with a linker peptide containing a TEV-protease recognition site. The TEV-protease recognition site is ENLYFQ-G [30,31], with the cleavage site between the glutamine and glycine amino acids. The *B*. *subtilis DOW01* strain used here contains an inducible TEV-protease (Table 1). The TEV-protease gene under control of the xylose inducible Pxyl promoter was amplified from BSG104 (primers constructTEV_FW & constructTEV_REV), inserted in pDG1664 and integrated in the *thrC* locus of the *B*. *subtilis* genome [32].

After cloning of the fluorophores into pDOW01, the resulting constructs were transformed and integrated in the *amyE* locus of *B*. *subtilis*. To facilitate transformation, *B*. *subtilis* was made naturally competent as described before [33]. Single copy, double recombination of the constructs was verified by the lack of (alpha)-amylase activity on LB starch plates.

### Western Blotting

An overnight culture of *B*. *subtilis* was diluted in fresh medium to an approximate OD_600_ of 0.03 and grown for 2h at 37°C, while shaking at 225 rpm. After induction with 0.1 mM IPTG, the cultures were split into two equal volumes, and 1% (w/v) xylose was added to one part to induce the TEV-protease. Cells were grown for 2 more hours at 37°C and 2 ml of cell culture was centrifuged (1 min, 10.000 rpm) and resuspended in 200 μl 50 mM Tris-Cl pH 7.4. Cell lysis was achieved by adding a small spatula tip of glass beads (<106 microns, Sigma) to the mix, followed by two times one minute mini-bead beating (Mini-Beadbeater-16, Biospec products). After centrifugation (2 min, 10.000 rpm) the supernatant was carefully transferred to clean tubes and stored at -20°C. 30 μl supernatant supplemented with SDS-loading buffer was boiled at 80°C for 10 minutes and loaded on a 12% SDS-PAGE gel. After completion of electrophoresis the gel was transferred to a PVDF Western Blotting membrane (Roche. One hour at 80 mA), followed by blocking with 5% (w/v) skim milk (Oxoid) in PBST (58 mM Na_2_HPO_4_, 17 mM NaH_2_PO_4_, 68 mM NaCl, 0.1% (v/v) Tween20, pH 7.3) overnight at 4°C. The PVDF-membrane was washed three times 15 minutes in PBST and incubated with PBST with 5% skim milk and a 1:10.000 dilution of anti-GFP (rabbit serum, Invitrogen Molecular Probes) for two hours at room temperature. The membrane was washed three times 15 minutes in PBST and incubated with PBST supplemented with 1:5.000 goat-anti-rabbit Ig-Horseradish Peroxidase (Amersham Biosciences) for 1.5 hour at room temperature. Subsequently, the membrane was washed three times, gently dried with tissue papers and incubated for two minutes with 2 ml of ECL detection reagent (GE Healthcare). Signal visualization was done with a Molecular Imager ChemiDoc XRS+ (BioRad).

### Fluorescence microscopy

#### Microscope specification

Microscope imaging for sensitized emission experiments was done using a Personal DeltaVision microscope system (Applied Precision, Issaquah, USA), with Softworx 3.6.0 software. The microscope was equipped with an Olympus IX71 inverted microscope body, a 100x phase contrast objective (Olympus PlanApo 1.40 NA), a CoolSNAP HQ2 camera (Princeton Instruments), a 300W Xenon light source, and filters for imaging of CFP (ex. 430/24 nm; em. 472/30nm), GFP (ex. 470/40 nm; em. 525/50 nm), and mCherry (ex. 572/35 nm; em. 632/60 nm) from Chroma. For Cerulean and Venus the CFP/YFP/mCherry polychroic mirror was used (Chroma, 460–500, 525–575, 590–680 nm range) and for GFP, mCherry, mKate2, and tagRFP the GFP/mCherry polychroic mirror was used (Chroma, 400–470, 490–570, 580–630 and 640–730 nm range). Images were captured using 0.2s light exposure with 32% light transmission for every combination of filters. The FRET channel was set in the software by using the CFP or GFP excitation filter and the GFP or mCherry emission filter.

### Strain preparation and protein overexpression

LB medium was inoculated from -80°C *B*. *subtilis* stocks and grown overnight at 37°C. The next morning the cultures were diluted 1 to 50 to an approximate OD_600_ of 0.03 in fresh LB medium and grown for two hours at 37°C at 225 rpm. The cells were induced with 0.1 mM IPTG and one part of the culture containing the two fluorophores was transferred into a new bottle containing 1% (w/v) xylose to induce the TEV-protease. After an additional two hours of incubation, cells were transferred to a microscope slide containing 1% (w/v) agarose to immobilize the cells and fluorescence intensity was measured with the wide-field microscope described above for FRET detection via sensitized emission. The same sample preparation method was applied for the FLIM experiments described below.

### Sensitized emission – Strain preparation for time-lapse

Time-lapse microscopy was done as described previously [34]. Briefly, LB medium was inoculated from -80°C stocks and grown for 8 hours at 37°C and 225 rpm. Subsequently, the culture was diluted 100 times in chemically defined medium (CDM; supplemented Spizizen’s salt [35], per liter: 2 g (NH_4_)_2_SO_4_, 14 g K_2_HPO_4_, 6 g KH_2_PO_4_, 1 g Na_3_citrate.2H_2_O, 0.27 g MgSO_4_.7H_2_O, 20 mg casamino acids (Formedium), 20 mg L-tryptophan and 5 g D-fructose) and grown overnight at 37°C, 225 rpm. The next morning the culture was diluted to an OD_600_ of 0.08 in fresh CDM, grown for 5–7 hours at 37°C and 225 rpm to an OD_600_ of approximately 0.7. Now (0.35/OD)*250 = 125 μl of cells were diluted in 500 μl fresh medium and 2 μl was transferred to a slide with 1.5% low-melting point agarose (Sigma) in CDM and 0.1 mM IPTG. When the TEV-protease gene should be expressed 1% (w/v) xylose was added to the slide medium as well. To make sure that the fluorophores were present right from the start of the time-lapse experiment, IPTG (final concentration 0.1 mM) was added to the liquid culture one hour before transfer to the agarose slide. And in case the TEV-protease should be expressed also 1% (w/v) xylose was added to the liquid medium. The agarose slide was divided in three columns, separated by air cavities; one for *B*. *subtilis DOW5* (GFP only), one for *B*. *subtilis DOW13* (tagRFP only), and one for *B*. *subtilis DOW23* (GFP-tagRFP).

Time-lapse was done for 16 hours with a 15 minute interval. Of every strain the same four pictures were taken: phase contrast (GFP transmission light), FRET (GFP excitation, mCherry emission), donor (GFP excitation and emission), and acceptor (mCherry excitation and emission). In all cases the light exposure time was 0.2s and the light transmission was 32%. The microscope setup was the same as above except for the light source, which was now solid state TruLight Illumination (Applied Precision, Issaquah, USA). The microscope had a software-controlled stage to visit selected points routinely during a time-lapse experiment and the DeltaVision UltimateFocus was used to keep cells in focus.

### Sensitized emission – Data analysis

Three biologically independent samples were used to obtain a total of eight images, necessary to do the FRET detection via sensitized emission and its corrections [6]: *B*. *subtilis* cells with only the donor, only the acceptor and with the donor and acceptor (Table 4). These samples with only one of the fluorophores are necessary to calculate the correction factor for the donor in the acceptor channel and vice versa, and to correct for bleedthrough (i.e. non-specific excitation and emission events in the partner fluorophore filter channels).

**Table 4:** The eight images required for sensitized emission FRET.

Symbol	Sample	Excitation filter	Emission filter
a	Donor only	Donor	Donor
b	Donor only	Donor	Acceptor
c	Acceptor only	Donor	Acceptor
d	Acceptor only	Acceptor	Acceptor
d2	Acceptor only	Donor	Donor
e (D)	Donor and Acceptor	Donor	Donor
f (S)	Donor and Acceptor	Donor	Acceptor
g (A)	Donor and Acceptor	Acceptor	Acceptor

Table was redrawn based on a table from the W.M. Keck Center for Cellular Imaging [46]. The wavelengths for the filters are specified in the fluorescence microscopy section.

Here, FRET was performed with fluorescent proteins that were linked to each other by a TEV-protease cleavage site. Induction of the TEV-protease will cleave the fluorophores apart and is expected to result in a lower FRET efficiency.

ImageJ (http://rsb.info.nih.gov/ij/index.html) was used to measure the pixel intensities of the *B*. *subtilis* cells and Microsoft Excel to process the data. First, the background pixel intensity was subtracted from the cellular pixel intensity and four correction factors were calculated to correct for the spectral overlap (Eqs 1–4). The letters in Eqs 1–6 [6] refer to the symbols in Table 4.

α=d2d (Eq 1)

γ=cd (Eq 2)

δ=d2c (Eq 3)

β=ba (Eq 4)

α corrects for the acceptor fluorescence in the donor channel, γ is the correction for the acceptor excitation efficiency by donor excitation light, δ corrects for the sensitized emission back into the donor channel and β is the correction for the donor fluorescence in the acceptor channel [6]. These factors were used to correct for bleedthrough of the fluorophores.

The FRET was calculated with Eq 5 [6] from the sensitized emission of the acceptor fluorophore in image S (Table 4). Subtraction of βD from S removes the donor contribution to the acceptor channel, subtraction of (γ-αβ)A is necessary to correct for direct acceptor excitation, and the image is scaled by dividing by 1-βδ.

$$\text{FRETsensitized emission}=\frac{\text{S}-\beta \text{D}-\left(\gamma -\alpha \beta \right)\text{A}}{1-\beta \delta }$$ (Eq 5)

To calculate the FRET efficiency, the sensitized emission from Eq 5 is divided by the acceptor fluorescence intensity, A (Eq 6 [6]). The obtained FRET efficiency, Ea, is independent of fluorescence intensities, which can vary over time due to protein expression levels.

FRETefficiency,Ea=FRETsensitized emission/A (Eq 6)

### Frequency Domain Fluorescence Lifetime Imaging Microscopy

The frequency domain MEM-FLIM system used (Lambert Instruments B.V.)[20], consists of a multi-LED light source containing 3W LEDs with peak intensities at 446 nm (for Cerulean) and 469 nm (for GFP), a signal generator and a prototype directly modulatable CCD camera. The MEM-FLIM system was mounted on a Nikon Eclipse Ti inverted microscope with a 100x oil phase contrast objective (1.40 NA). The filter combinations used for imaging Cerulean were ex. 436/20 nm; em. 480/40nm and for GFP em. 480/30 nm; ex. 535/40 (Nikon). Erythrosine B (Sigma-Aldrich 87613), a fluorescein derivative, with a lifetime of 0.086 ns was used as a reference. Erythrosine B was dissolved in H_2_O and used with a concentration that matched the brightness of the samples. The MEM-FLIM system was operated using LI-FLIM software version 1.2.24 (Lambert Instruments B.V.).

Phase contrast images were taken using transmission light and 0.1s exposure time. FLIM data was collected using 0.7s exposure time and modulated LED light for excitation. Fluorescence lifetime data was collected using a modulation frequency of 40 MHz. For obtaining single cell fluorescence lifetime data the additional 1.5x magnification on the Nikon Microscope was used and the exposure time for collecting fluorescence images was extended to 1.5s. The LI-FLIM software was used for calculating the fluorescence lifetimes from phase shift data, see Zhao *et al*. [20] for details.

### Acceptor photobleaching

Acceptor photobleaching was performed as described previously [36]. Briefly, for all strains the following pictures were made: three pictures before bleaching with the following filter settings: phase contrast, donor excitation and emission, and acceptor excitation and emission, now the acceptor was bleached for one minute at 100% light transmission using the mCherry filters, and then three pictures after bleaching were made with the following filter settings: phase contrast, donor excitation and emission, and acceptor excitation and emission. ImageJ was used to determine the fluorescence intensities of the donor before and after bleaching the acceptor. Pictures were taken from the donor-only strain (*DOW05*), and from the strain with donor and acceptor (*DOW23*) with and without induction of the *tev* protease encoding gene.

The FRET efficiency can be calculated with Eq 7 [36].

$$\text{FRETefficiency},\;\text{E}=\left(I_{DA*}-I_{DA}\right)/I_{DA*}$$ (Eq 7)

I_DA_ is the donor fluorescence intensity in presence of the acceptor and I_DA*_ is the donor fluorescence intensity after photobleaching the acceptor.

## Results

### Single cell observations of FRET detected via sensitized emission

**Fig 2:**
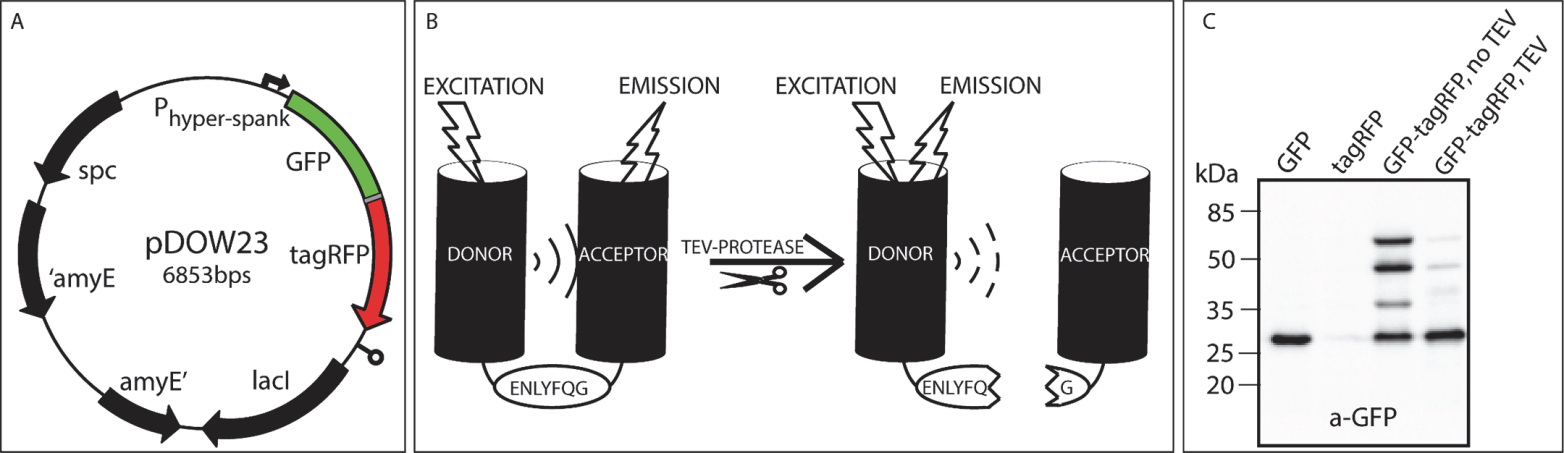
(A) The *amyE* integration vector pDOW23 with FRET-pair GFP-tagRFP. (B) Schematic representation of two fluorescent proteins and the linker containing the TEV-protease recognition site (ENLYFQG). (C) A Western Blot to show the cleaving of coupled fluorophores by the TEV-protease. GFP protein was visualized by chemiluminescence with GFP-antibodies. The lanes contain cell free extract from the following strains: lane 1, *DOW05* (*thrC::Pxyl-tev amyE::gfp*), lane 2, *DOW13* (*thrC::Pxyl-tev amyE::tagRFP*), lane 3, *DOW23* (*thrC::Pxyl-tev amyE::gfp-tagRFP*) from a culture without induction of the *tev* protease gene and lane 4, *DOW23* (*thrC::Pxyl-tev amyE::gfp-tagRFP*) in which the *tev* protease gene was induced with 1% (w/v) xylose. Predicted sizes for GFP and tagRFP monomer are 27 kDa, and the complex 55 kDa.

The aim of this work was to identify the best FRET-pair and to perform FRET at the single cell level in *B*. *subtilis* using fluorescence microscopy. Therefore, the suitability of various fluorescent proteins (FPs) for FRET purposes in *B*. *subtilis* was tested by expressing them pairwise and covalently linked. The FPs tested here were Cerulean (a cyan FP [25]), Venus (a yellow FP [26]), and sfGFP(Sp) [27] as donor and tagRFP (a red-orange FP, Evrogen), mCherry (a red FP [28]), and mKate2 (a far-red FP, Evrogen) as acceptor. The respective genes were cloned in the *amyE* locus of *B*. *subtilis* under control of the IPTG-inducible P_*hyper-spank*_ promoter, by using the newly constructed *amyE* integration vector pDOW (Fig 1), which allows for efficient BglBrick [23] assembly (Fig 2A).

Measuring both high and low intracellular FRET efficiency is essential for benchmarking the methodology used in this study. Covalently linked pairs of fluorophores were constructed, using a linker that contains a TEV protease recognition site (Fig 2B). The functionality of the TEV-protease was tested before the search for the best FRET pair was started, to ensure that the protease is able to separate the two fluorophores from each other. Induction of TEV protease should induce uncoupling of the FRET pair, which results in loss of sensitized emission of the acceptor. Western blotting with GFP specific antibodies was performed to visualize the presence and size of the GFP containing proteins. As shown in Fig 2C, lane 3, the GFP-tagRFP synthetic dimer was readily produced. Upon induction of the *tev* gene, the heterodimer was efficiently cleaved into the monomers GFP and tagRFP (lane 4). Expression of the *tev* gene was a bit leaky, resulting in the presence of monomeric GFP without induction of *tev* expression (lane three). The band around 37 kDa in lane three is assumed to be a degradation product of the dimer. Overall, these results show that induction of the *tev*-protease gene results in an efficient separation of the FP pair (Fig 2C, lane 4).

**Fig 3:**
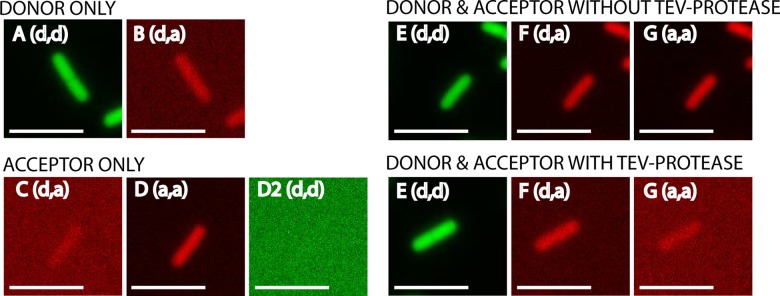
Fluorescence intensities of single cells for the FRET-pair GFP-tagRFP. Microscope excitation and emission filter settings are shown between brackets (d = donor, a = acceptor). For donor the filters (excitation, emission) were: GFP, GFP; for acceptor: mCherry, mCherry; and for FRET the filters were: GFP, mCherry. In all cases a GFP/mCherry polychroic mirror was used (400–470, 490–570, 580–630 and 640–730 nm range). A and B are cells where only donor fluorophore is present (GFP). C, D and D2 are cells where only acceptor fluorophore is present (tagRFP). E, F and G (upper panel) are cells where donor-acceptor fluorophore (GFP-tagRFP) are coupled and TEV-protease is not induced. E, F and G (lower panel) are cells where donor-acceptor (GFP-tagRFP) are uncoupled by induction of TEV-protease. The same signal scaling is used for all images. Note that the signals are false colored (GFP: green, tagRFP: red). Scale bar is 5 μm.

The process of data acquisition and analysis to calculate the FRET efficiency is shown with single cell images of the FRET pair GFP-tagRFP (Fig 3 and Table 4). First, cells with only donor and only acceptor were imaged under the microscope in three channels (Fig 3A–3D2). Next, cells with donor and acceptor (the FRET pair) were imaged in the same three channels both with and without induction of TEV-protease (Fig 3E–3G). Note the significant decrease in acceptor fluorescence (Fig 3F) in the presence of TEV-protease. The contributions of donor emission in the acceptor channel (Fig 3B) and the excitation of the acceptor by donor excitation (Fig 3C) were very small, but nevertheless these contributions need to be taken into account, because this light in the FRET channel is not due to sensitized emission of the acceptor. After measuring the fluorescence intensities from all cells (Fig 3) the FRET efficiency measured via sensitized emission was calculated with Eq 6.

FRET efficiencies of the different fluorophore pairs detected with sensitized emission are shown inTable 5. The following criteria were used to select the most suitable FRET pair from the various combinations of FPs: first, the signal to noise levels of the separate fluorescent proteins should be high (Table 6), so localization and dynamics of individual proteins fused to a given fluorescent protein can be studied. Second, the difference in FRET efficiency between the covalently bound and cleaved fluorophores should be high. The highest signal to noise levels for the individual fluorophores were observed in the case of GFP, mKate2 and tagRFP (Table 6). Therefore, GFP was selected as FRET donor in subsequent protein-protein interaction experiments. The best acceptors were tagRFP and mKate2. The quantum yields were 0.48 versus 0.40 [29,37] and the relative brightness was 142 versus 74 for tagRFP and mKate2, respectively (as percentage of EGFP) [29]. Both fluorophores are monomeric [29], but the higher quantum yield of tagRFP will make protein interaction studies with the tagRFP easier for FRET analysis via sensitized emission since more of the donor resonance energy transfer to the acceptor will result in emission from the acceptor [38].

**Table 5:** The FRET efficiency Ea of the different FRET pairs, measured via Sensitized Emission.

		FRET efficiency, Ea		
	R_0_	Donor-Acceptor covalently bound	Donor-AcceptorCleaved by protease	Difference
Cerulean-Venus	5.3^a^	0.02 +/- 0.00	0.01 +/- 0.00	0.01
GFP-mCherry	5.28[38]	0.05 +/-0.03	0.02 +/- 0.00	0.03
GFP-mKate2	~5.31^b^	0.14 +/- 0.02	0.00 +/- 0.03	0.14
GFP-tagRFP	5.74[38]	0.22 +/- 0.09	0.01 +/- 0.06	0.21
Venus-mCherry	5.7^a^	0.03 +/- 0.02	0.01 +/- 0.00	0.02

^a^ http://www.microscopyu.com/tutorials/java/fluorescence/fpfret/index.html

^b^ Calculated based on the GFP, mKate2, and tagRFP spectra from www.evrogen.com. For formulas see reference [39,44]. The spectral overlap of GFP-tagRFP and GFP-mKate2 are very similar (Evrogen spectra), but the extinction coefficient ε_A_ of mKate2 is smaller than the ε_A_ of tagRFP (62,500 vs. 100,000 M^-1^ cm^-1^). Multiplying the spectral overlap J(λ) of GFP-tagRFP with 0.625 resulted in a calculated R_0_ of 5.31 for GFP-mKate2.

**Table 6:** Fluorescence intensities of *B*. *subtilis* cells with the various fluorescent proteins.

	Fluorescence intensity						
	Donor only		Acceptor only		Donor and acceptor (FRET)		
	(d,d)^a^	(d,a) ^a^	(a,a) ^a^	(d,a) ^a^	(d,d) ^a^	(d,a) ^a, b^	(a,a) ^a^
Cerulean	4.86±0.86	2.42±0.45					
Venus			373.16±40.85	3.81±0.47			
Cerulean-Venus					4.18±1.71	**9.29**±**2.68**	266.44±43.12
Cerulean, Venus cleaved					3.73±0.67	**5.24**±**0.89**	214.32±15.61
GFP	432.39±85.25	11.23±2.47					
mCherry			57.00±22.97	2.14±0.60			
GFP-mCherry					583.88±33.12	**29.31**±**5.39**	165.42±14.77
GFP, mCherry cleaved					555.99±18.56	**21.61**±**1.61**	127.14±23.68
GFP	471.13±20.20	13.98±1.62					
mKate2			77.40±16.53	4.94±1.06			
GFP-mKate2					483.29±55.88	**33.74**±**5.99**	98.90±21.60
GFP, mKate2 cleaved					536.91±114.16	**20.70**±**4.79**	86.11±31.93
GFP	421.92±84.29	11.53±1.73					
tagRFP			41.06±7.36	3.82±0.69			
GFP-tagRFP					572.89±12.98	**38.21**±**9.20**	72.16±13.73
GFP, tagRFP cleaved					563.96±57.18	**19.44**±**1.31**	36.28±25.79
Venus	163.11±16.79	15.82±7.10					
mCherry			81.55±6.49	4.62±1.39			
Venus-mCherry					190.35±56.06	**33.11**±**11.96**	190.63±1.20
Venus, mCherry cleaved					138.84±24.14	**22.76**±**9.61**	144.52±21.69

The column with the FRET channel, the most important part of the data, is highlighted. Fluorescence intensities were measured with three different microscope filter settings for excitation and emission filters: donor,donor; donor,acceptor; and acceptor,acceptor (as indicated by the letters between brackets^a^. d = donor, a = acceptor). ^b^Sensitized emission is determined from this sample and these filter settings. The values here are a triplicate of the average intensity of 50 cells. The standard deviations are shown as well.

Based on the criteria of high signal to noise fluorescence and a large difference in FRET efficiency between the bound and cleaved fluorophores, GFP-tagRFP was chosen as the best FRET pair in *B*. *subtilis* (Table 5). Both the fluorescence intensities and the FRET efficiency of the Cerulean-Venus, the GFP-mCherry, and the Venus-mCherry combinations were much lower than of the GFP-mKate2 or GFP-tagRFP combinations; therefore these pairs were excluded from further analysis.

### FRET detection via sensitized emission can be confirmed by acceptor photobleaching

To support the above-presented FRET detection method via sensitized emission, an acceptor photobleaching experiment on the GFP-tagRFP fluorophore pair was performed. FRET efficiency can be determined via acceptor photobleaching [36]. When FRET occurs, the acceptor molecule quenches the donor fluorescence (resulting in decreased donor fluorescence), but when the acceptor is destroyed by photobleaching it cannot quench the donor anymore, so increased donor fluorescence can be detected.

To specifically photobleach tagRFP, we placed live cells under the microscope and excited with 572/35 nm with 100% of the output of solid state TruLight Illumination for one minute. This resulted in a decrease of 35% of the tagRFP fluorescence.

Indeed, using the GFP-tagRFP pair, the donor (GFP) emission was lower when FRET occurs than when GFP and tagRFP are uncoupled by TEV-cleavage. The pre-bleach fluorescence intensities of GFP were: 362 AU without TEV protease vs. 437 AU when TEV was produced. Moreover, after acceptor photo bleaching, an increase in donor fluorescence was observed when the GFP-tagRFP was coupled: the fluorescence intensities of GFP were 410 AU (13% increase) when GFP-tagRFP was coupled vs. 429 AU (1.8% decrease) when GFP-tagRFP was uncoupled. Acceptor photobleaching increases the GFP fluorescence with 13% when the fluorophores were linked to each other. When the fluorophores were uncoupled by the TEV-protease, the donor fluorescence was approximately the same before and after photobleaching (437 vs. 429), which is a good control for this method.

The FRET efficiency E (see Eq 7) is $\left(I_{DA*}-I_{DA}\right)/I_{DA*}$ = (410–362)/410 = 0.12 for GFP-tagRFP in this acceptor photobleaching experiment.

In total, we showed that the GFP-tagRFP pair can be efficiently used as a FRET pair for protein interactions in live *B*. *subtilis* cells.

### FRET efficiency dynamics in time-lapse experiments

**Fig 4:**
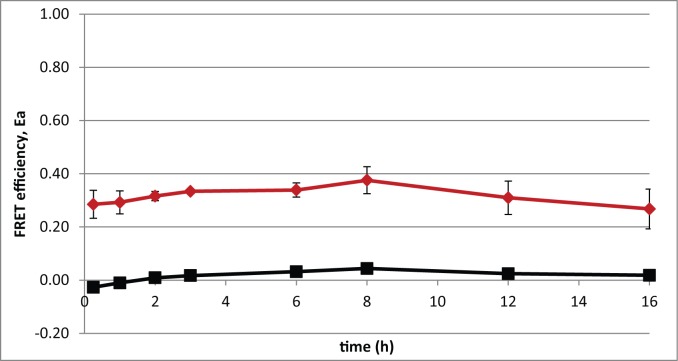
The FRET efficiency, Ea, was determined over time with a fluorescence microscopy time-lapse experiment. The covalently bound GFP-tagRFP, e.g. no TEV-protease, results in a high FRET efficiency (red line) and when the GFP-tagRFP is uncoupled by inducing the TEV-protease encoding gene, it results in a low FRET efficiency (black line). Error bars show the standard deviation of three replicate experiments. At least 50 single cells were analyzed at each time point.

This study focusses on finding appropriate FRET-pairs for studying temporal behavior of protein-protein interactions. The GFP-tagRFP FRET pair was used in a time-lapse experiment to determine if the FRET efficiency is stable over time. Both covalently bound and TEV-protease treated GFP-tagRFP produce a constant FRET efficiency (Fig 4). FRET-efficiency altering processes like unequal protein degradation was hereby excluded. Any FRET-efficiency dynamics found in future protein-protein interaction experiments can be attributed to the given protein-protein interactions.

### FRET-FLIM

The fluorescence lifetime of GFP on its own was 2.56 ns (Table 7). When GFP was coupled to an acceptor, the fluorescence lifetime was reduced, i.e. 2.22 ns for GFP-tagRFP, and when the GFP and the acceptor were uncoupled by expression of TEV-protease the GFP fluorescence lifetime increased again (Table 7). However, the fluorescence lifetime of GFP in the uncoupled FRET pair did not increase back to the situation of GFP only, which might indicate that the cleavage of the fluorophores was not 100%.

**Table 7:** The FRET efficiency Ea of the different FRET pairs, measured with FLIM.

	Fluorescence lifetime (ns)		
	Donor-Acceptor covalently bound	Donor-AcceptorCleaved by protease	Ea
GFP-mCherry	2.37 +/- 0.03	2.47 +/- 0.01	0.04
GFP-mKate2	2.24 +/- 0.02	2.51 +/- 0.03	0.11
GFP-tagRFP	2.22 +/- 0.02	2.51 +/- 0.01	0.11

The fluorescence lifetime of GFP_only is 2.56 ns. The fluorescence lifetimes shown here are calculated with the LI-FLIM software from the average of all pixels in five regions of interest filled with a monolayer of cells.

The FRET efficiency can be calculated from the fluorescence lifetimes with Eq 8 [39].

$$\text{FRET Efficiency}\;E=1-\frac{\tau_{DA}}{\tau_{D}}$$ (Eq 8)

where τ_DA_ is the fluorescence lifetime of the donor in presence of an acceptor and τ_D_ is the fluorescence lifetime in the absence of an acceptor. The highest FRET efficiencies were 11% and were obtained for GFP-mKate2 and GFP-tagRFP, the FRET efficiency for GFP-mCherry was only 4%. The Cerulean fluorescence lifetime could not be determined, because of technical limitations (non-appropriate filters on the MEM-FLIM mounted microscope).

**Fig 5:**
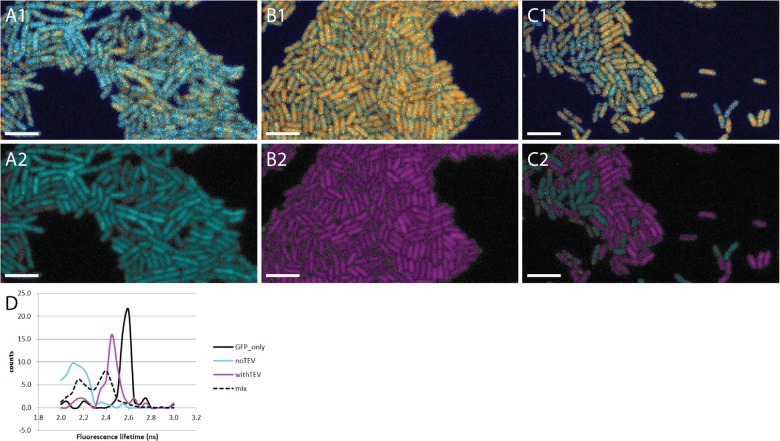
Single cells FLIM measurements. (A1) *B*. *subtilis* cells are shown where the GFP-tagRFP fluorophores are linked. (B1) *B*. *subtilis* cells are presented where the GFP and tagRFP fluorophores are cleaved apart. (C1) *B*. *subtilis* cells where GFP-tagRFP fluorophores are linked are mixed in a 1:1 ratio with *B*. *subtilis* cells where the GFP-tagRFP fluorophores are cleaved apart; resulting in a mix of cells with either short GFP fluorescence lifetime due to quenching by tagRFP or long GFP fluorescence lifetime. Visualization of cells in A1, B1, C1 was done with a Look-Up-Table from LI-FLIM. A2, B2 and C2 present the same cells, but now a Matlab script was used to categorize the cells into two categories: cells with short GFP lifetimes are shown in cyan and cells with long GFP lifetimes are shown in magenta. (D) fluorescence lifetime based histogram of the cells described in A2-C2, black, cyan, magenta and dotted lines present GFP_only, linked fluorophores, cleaved fluorophores and a mix of the two populations, respectively. Scale bar is 5 μm.

Cells containing GFP-tagRFP were used to study the usability of the prototype MEM-FLIM system for FRET-FLIM measurements at the single bacterial cell level (Fig 5). In the top part of Fig 5 cells with coupled fluorophores, cells with uncoupled fluorophores or a mix of cells with coupled and uncoupled fluorophores were false-colored with a look-up-table from the LI-FLIM software. Using a Matlab script for automated cell sorting, these cells were categorized into two groups based on fluorescence lifetime values; cells with short lifetime were false-colored cyan and with long lifetime were false-colored magenta; threshold value was set to 2.3 ns (Fig 5A2–5C2). This script was also used to make a fluorescence lifetime based histogram (Fig 5D). The histogram confirms that the cells from Fig 5C2 contained cells with short and long fluorescence lifetime. This showed that the MEM-FLIM prototype allows single bacterial cell FLIM and can resolve inter-individual fluorescence lifetimes.

## Discussion

Intermolecular FRET analysis allows *in vivo* examination of protein-protein interactions. It has been successfully applied for studying the sensor kinases CitA and DcuS in *E*. *coli* [40] and the Fts division proteins in *E*. *coli* [15]. The proteins from the competence machinery in *B*. *subtilis* have been studied via acceptor photobleaching [16] and the chemotaxis pathway in *E*. *coli* has been studied extensively with acceptor photobleaching as well [41], but acceptor photobleaching does not allow examination of the dynamics.

Here we studied which FRET-pair would be a suitable candidate for *in vivo* FRET analysis in *B*. *subtilis*. FRET detected via sensitized emission showed that, out of the pairs tested, the GFP-tagRFP pair is the best candidate for FRET purposes in *B*. *subtilis*, based on the relative brightness and the quantum yield of tagRFP (see also results section). Earlier work showed the suitability of GFP-tagRFP and GFP-mCherry FRET-pairs in HeLa cells [38,42]. However, the observed FRET efficiency in *B*. *subtilis* is low for GFP-mCherry using both sensitized emission and FLIM (Tables 5 and 7), despite the fact that the spectral overlap between GFP and mCherry is high as well as the fluorescence intensities. In case of sensitized emission the low FRET efficiency for the GFP-mCherry combination could also be the result of the calculation method used in our study (Eq 6). Division by A – the acceptor fluorescence intensity – results in lower FRET efficiency for GFP-mCherry, because A is much higher for GFP-mCherry than for GFP-tagRFP or GFP-mKate2 (Table 6). However, FLIM measurement data is independent of intensities and the FRET-FLIM data confirms the data from the sensitized emission experiments (Tables 5 and 7). In both cases GFP-mKate2 and GFP-tagRFP are the best two FRET pairs.

GFP-mCherry is often used as a FRET pair in interaction studies with high FRET efficiencies. In this study it might be that the properties of the linker prevent proper orientations of both fluorophores resulting in poor FRET efficiencies for this FRET-couple. FRET efficiency is dependent on three criteria for obtaining FRET. Of those criteria only the spectral overlap is independent of the construct used. The fluorophore distance and relative orientation of the donor and acceptor molecules depend on the linker sequence. Therefore, the FRET efficiencies reported here reflect the situation with the TEV-protease cleavable linker. FRET-efficiencies in earlier work range from 4 to 46% [15,16,40,43].

Individually, the fluorescent proteins are efficiently produced (Table 6) and, when linked together, the GFP-tagRFP pair has the highest FRET efficiency. The benefit of red-shifted FRET-pairs is in accordance with earlier work [38,44], and one possible explanation is the larger Förster radius [44].

The FRET-FLIM set-up used here allows measurements of FRET-efficiency on a single bacterial cell level (Fig 5). This shows the potential of this system for the study of heterogeneity in protein-protein interactions. At this point, the prototype CCD-sensor has limited sensitivity, only allowing single bacterial cell FLIM with highly expressed FRET pairs and is therefore not yet widely applicable for studies in bacteria. However, when fusion proteins are put under control of strong promoters, relevant data might be obtainable, even for normally low-expressed proteins. Alternatively, improved systems could be incorporated in existing microscopy set-ups allowing fast FRET readouts during e.g. time lapse microscopy or microfluidic experiments for the study of protein-protein interaction dynamics.
